# Silence and related symptoms in children and adolescents: a network approach to selective mutism

**DOI:** 10.1186/s40359-022-00956-9

**Published:** 2022-11-16

**Authors:** Felix Vogel, Julian Reichert, Christina Schwenck

**Affiliations:** 1 Department of Special Needs Educational and Clinical Child and Adolescent Psychology, Justus-Liebig-University of Giessen, Otto-Behaghel-Straße 10 E, 35394 Giessen, Germany; 2 Department of General Internal Medicine and Psychosomatics, University Hospital Heidelberg, Heidelberg, Germany

**Keywords:** Adolescents, Anxiety, Children, Network analysis, Selective mutism, Symptoms

## Abstract

**Background:**

Silence in certain situations represents the core symptom of selective mutism (SM). However, it is unclear what additional symptoms are part of this disorder. Although knowledge of symptoms is essential for diagnostics and intervention, to date, only scarce research exists on circumscribed symptoms of SM. Given the large overlap between SM and social anxiety disorder (SAD), it remains also unclear which symptoms can differentiate both disorders.

**Methods:**

A network analysis of potential symptoms of SM was performed based on a mixed sample of N = 899 children and adolescents with and without indication of SM (n = 629 with silence in certain situations). In a preliminary analysis, we demonstrated that children with and without silence in certain situations do not differ with respect to their network structure, justifying an analysis on the entire mixed sample. Possible communities (symptom clusters) within the network and thus potential latent variables were examined, and symptoms were analyzed in terms of their centrality (the extent to which they are associated with other symptoms in the network). To investigate the differentiability of symptoms of the SM network from symptoms of SAD, we computed a network that additionally contains symptoms of SAD.

**Results:**

In the resulting network on symptoms of SM, silence was, as expected, the symptom with the highest centrality. We identified two communities (symptom cluster): (1) symptoms associated with the fear response of freezing, (2) symptoms associated with speech production and avoidance. SM network symptoms and SAD symptoms largely formed two separate symptom clusters, with only selectivity of speaking behavior (more talkative at home and taciturn or mute outside the home) falling into a common cluster with SAD symptoms.

**Conclusions:**

Silence appears to have been confirmed by analysis as a core symptom of SM. Additional anxiety-related symptoms, such as avoidance behavior or motor inhibition associated with freezing, seem to co-occur with silence. The two communities of SM potentially indicate different mechanisms of silence. The symptoms of SM appear to be distinguishable from those of SAD, although there seems to be overlap in terms of difficulty speaking in situations outside the home.

**Supplementary Information:**

The online version contains supplementary material available at 10.1186/s40359-022-00956-9.

## Introduction

Circumscribed symptoms are essential for a reliable and valid diagnosis of mental disorders [1] and represent important targets for interventions [2]. In this context, empirical research on symptoms reveals which symptoms in specific mental disorders can be considered as representative and thus important for diagnosing the disorder [1, 3, 4]. Furthermore, implications for important targets of treatment might be drawn from analyses regarding the significance of circumscribed symptoms [2]. Although research at the symptom level is still at an early stage [5, 6], it is a promising approach to extend the understanding of a mental disorder.

### Symptoms of selective mutism in the DSM-5

Silence in certain social situations (e.g., in school) with unimpaired speech production in other social situations (e.g., at home) represents the core symptom of selective mutism (SM) [7]. However, the diagnostic criteria of DSM-5 do not define any other circumscribed symptom for SM beyond silence. This is surprising, as SM was classified as an anxiety disorder with the introduction of the DSM-5, and for all other anxiety disorders, additional symptoms beyond the core symptom are described in the diagnostic criteria. For example, in social anxiety disorder (SAD), the DSM-5 defines that a marked fear reaction (in children expressed by crying, tantrums, freezing, or clinging, shrinking, or failing to speak in social situations) and avoidance behavior (or endurance of the situation with intense fear) occur together with the core symptom of a marked fear of scrutiny by others. Some of these anxiety-related symptoms (e.g. social fears or clinginess) are indeed outlined in the associated features section of SM in the DSM-5. Given that SM shares numerous features with anxiety and social anxiety in particular [8] and has a high co-incidence with other anxiety disorders (especially SAD) [9], it seems reasonable to assume that additional anxiety-related symptoms occur together with silence as well. However, the section on associated features of SM in DSM-5 comprises also non-anxiety-related symptoms such as externalizing behaviors and communication disorders. This reflects research findings that anxiety is not the central phenomenon in all children with SM and the symptom domains mentioned here may also be important [8, 10, 11]. However, little research has systematically examined the significance of possible circumscribed symptoms of SM and distinguished them from symptoms of other disorders (e.g. SAD). Given high rates of undetected cases in children with SM [12] and a long average time between the onset of SM and its clinical presentation [13], the investigation of additional potential symptoms of SM in order to improve detection of children with SM appears to be of central importance.

### Research on possible symptoms of SM

To date, little systematic research exists on the relevance of possible symptoms of SM. However, indirect evidence of possible symptoms that may be relevant to SM can be derived from various strands of research on SM. First, studies that directly asked children with SM or their parents about the relevance of clinical features of SM based on an open-ended response format or at the item level can be noted. For example, children and adolescents with SM were asked to report fear content and the frequency of cognitions [14]. In addition to the relevance of social fears and different fear-related cognitions, a subset of children and adolescents reported avoidance behavior and strong inhibition by fear as important clinical features [14]. In a study by Ford et al. [15], complementary to the absence of speech, items regarding shyness, social anxiety, and oppositional behavior were shown to be rated most frequently as relevant features. Furthermore, in the study by Remschmidt et al. [13], the most frequently reported features were pronounced anxiety and insecurity. Interestingly, lack of contact as well as psychomotor disturbances have been reported to be important clinical features of most participants. Even if these studies indicate the relevance of different potential symptoms of SM, they are based on descriptive data only, and no conclusion can be drawn about the relationship between the possible symptoms. Second, there are studies that have investigated the relationship between symptomatology of SM and various constructs assessed by validated questionnaires or interviews. Here, studies suggest the temperamental trait of behavioral inhibition (BI), which is expressed, for example, in terms of distress to novelty, shyness, and fear responses such as a strong inhibition in new environments or toward strangers [16], to be important in children with SM [17, 18]. Furthermore, it has been consistently shown that children with SM have, on average, clinically relevant levels of social anxiety [8]. However, latent profile analyses of children with SM indicate that only a minority of children with SM are exclusively characterized by elevated anxiety and that most children with SM exhibit other symptoms in addition to increased anxiety [10, 11, 19, 20]. Externalizing symptoms [20, 21], developmental delays including communication delays [20, 22], among others, also seem to be related to the symptomatology of SM. Given that these studies are based on questionnaire sum scores, it is unclear which specific feature of the construct might be particularly relevant in children with SM and how it might relate to other symptoms of SM. Third, quasi-experimental studies with children with SM may indicate potential symptoms of SM. In this context, a study based on behavioral observation showed that children with SM can be characterized not only by complete silence in social situations, but also seem to show reduced speech (e.g., lower frequency of speech or longer latency to respond) as well as a longer latency to initiate nonverbal communication [23]. A study based on eye-tracking showed that children with SM have reduced visual exploration, which also correlates with a non-validated parent report regarding frozen motor activity of their children [24]. Thus, features of the fear response of freezing such as inhibition of motor activity including vocal inhibition [25], might be important in children with SM. It was also found in this study that SM symptomatology was associated with a lower duration of fixation on the eye area of a social counterpart [24], suggesting that avoidance of direct eye contact may be a clinical feature of SM. Given the large overlap between SM and SAD and a debate about whether the two disorders are a common entity or two distinct entities [8], some quasi-experimental studies directly compared children with SM and SAD on different features. Children with SM and SAD did not differ with respect to most of the investigated features including attentional focus in social situations [24], fear-related cognitions [14], cortisol reactivity [26], autonomic response to social stress [27] or state anxiety in embarrassing social situations [23, 26, 28]. Interestingly, children with SM and SAD could be differentiated based on the score of the diagnostic scale (DS) of the Frankfurt Scale of Selective Mutism (FSSM), a validated questionnaire on SM, which asks for pathognomonic symptoms of SM [29]. However, again it is unclear whether all and which symptoms differentiate between the two disorders, as this result was based on sum score. Nevertheless, validated questionnaires for SM seem to be a good starting point for a detailed analysis of the relevance of possible symptoms of SM, as they can identify children with SM and differentiate them from children with SAD.

### Symptoms of SM derived from validated questionnaires

There are two disorder-specific validated screening questionnaires for SM [29, 30] from which possible symptoms of SM might be derived. The Selective Mutism Questionnaire (SMQ) has a meaningful factor structure as well as good reliability and construct validity, but it does not include circumscribed symptoms of SM [30]. Instead, it asks about the failure to speak in different situations and the resulting impairment. In contrast, the DS of the FSSM asks for core characteristics of SM [29]. Based on the core characteristics of SM contained in the DS, the FSSM provides clinical cut-off values with high sensitivity and specificity, which was shown by a receiver operator analysis [29]. The circumstance that the circumscribed characteristics of SM included in the DS differentiate between SM and children with other related disorders (SAD and internalizing disorders) indicates the relevance and specificity of these features for SM [29]. In addition to silence in certain situations, the DS of FSSM contains items about the selectivity of speaking behavior concerning the difference between speaking at home and outside, reduced speech (quiet, toneless or single words), noise suppression (e.g., sneezing), and an incapacity to communicate nonverbally in verbal situations. Furthermore, variations of motor inhibition in social situations (e.g. frozen-like movement, frozen facial expression) as well as avoidance behavior (avoidance of verbal situations and avoidance of eye contact) are included. Because previous research on SM symptoms either has examined only the sum scores of individual symptom domains or neglected the interrelationships among symptoms, a fine-grained analysis of circumscribed symptoms and their interactions appears to be of great importance.

### Network theory of psychopathology

Network theory of psychopathology is such an approach that provides a framework for examining the importance of circumscribed symptoms and their interaction for a mental health disorder. It is assumed that a network of causally connected symptoms (nodes of the network) activating and influencing each other (edges of the network) constitutes a mental disorders [2, 31, 32]. In contrast to assessing psychopathology using sum scores of validated questionnaires, which is associated with a loss of information [3], the network approach has the advantage of taking into account the significance and interplay of individual symptoms. It further provides the possibility to visualize the connections between these symptoms. Compared to a purely descriptive examination of the frequency of symptoms, the network approach offers the advantage of comparing the symptoms concerning their relevance and of examining their connection. Structures such as clusters of conceptually similar symptoms (or so-called communities) within the symptom network can be statistically detected, which allows for drawing conclusions about potential constructs or latent variables that are associated to the symptom clusters [33]. Additionally, the significance of a circumscribed symptom can be quantified in terms of its centrality, indicating a symptom’s connectivity and thus its relative influence on the other symptoms [34] and in terms of predictability, indicating how much of its variance can be explained by other symptoms of the network [35, 36]. Symptom networks with a high average predictability (e.g., anxiety disorders) are considered to be more strongly influenced by symptoms within the network, whereas symptom networks with a low average predictability are considered to be influenced more strongly by factors outside the network (e.g., environmental factors) [35, 36]. Hence, it is assumed that therapeutic interventions addressing symptoms of a strongly linked network also have a strong influence on the other symptoms. In addition to analyses within a symptom network of a single mental disorder, it is also possible to analyze how symptom networks of two different mental disorders are related [37]. Here it can be examined which symptoms represent connecting elements or so-called bridge symptoms between two disorders (based on centrality values). In this context, it has been shown that bridge symptoms play a crucial role in the spread of a mental disorder and thus in the development of comorbidities [37]. Symptom networks can be calculated in clinical as well as community or mixed samples as long as there is no qualitative difference concerning the symptom network between a clinical and a healthy group [38]. Under this condition, network analysis based on the network of healthy or mixed samples can inform about the symptom network of a clinical group [39]. While there is no such research in the context of SM, the network approach has already been used to empirically investigate the significance of circumscribed symptoms of other mental disorders [1, 39–42].

### Current study

The aim of the present work is to conduct a network analysis of symptoms potentially associated with SM. Symptoms included in the analysis were from a validated questionnaire on SM, which comprises pathognomonic features of SM and has a high discriminatory ability against SAD. Since to date, there is no network analysis in this context, the present study is largely exploratory. Here, we aim to (1) verify whether all symptoms included in the analysis form a positively linked network and can thus be considered to be related to the core symptom of SM, (2) investigate whether symptoms related in content form symptom clusters (so-called communities) within the network, (3) and explore which symptoms are most strongly connected with other symptoms of the network (centrality/strength) as well as which of them are able to explain most of the variance in other symptoms of the network (predictability). Here, we assume that the core symptom of SM (silence) has the highest centrality and predictability. We further (4) investigate whether the symptom network of SM can be differentiated from symptoms associated with SAD. This would give an indication of whether these are SM-specific symptoms or whether they could also be attributable to the strongly related disorder of SAD. Given that children with SM and children with SAD could be differentiated based on the sum score of symptoms of DS of FSSM [29], two separate symptom clusters of symptoms of SM and symptoms of SAD can be expected.

## Methods

### Procedure

The study at hand is based on data from four different projects in which symptomatology of SM was assessed by the Frankfurter Scale of Selective Mutism (FSSM). Full descriptions of the procedures of three of these studies (a–c) can be found here: (a) [14], (b) [28, 43]; (c) [24, 27]. The FSSM was (a) completed by n = 448 parents of children and adolescents aged 3 to 18 years in the first study, (b) n = 172 parents of children and adolescents aged 3 to 17 years in the second study and (c) n = 188 parents of children aged 8 to 12 years in the third study. Studies (a) and (b) were based on an online questionnaire that captured (a) fears related to SM and anxiety levels in different social situations and (b) characteristics that elicit symptomatology in SM. In the third study (c), the FSSM was also administered via online questionnaire, and then, a proportion of families was visited in their homes where attentional processes as well as psychophysiological responses in children with SM were investigated. The fourth study (d), not yet published, also consisted of an online questionnaire asking parents of children and adolescents with SM aged 3 to 17 years about possible symptoms in different situations. Here, the FSSM was assessed online in n = 91 parents.

### Participants

Overall, the total sample (see Table 1) of the present study consists of N = 899 individuals (female individuals: n = 598, 65.5%) with an age range of 3 to 18 years and an average age of M = 9.30 (SD = 4.22). Of these, n = 631 parents indicated that their children display the core symptom of SM (silence in certain social situations, indicated by M1 of FSSM, see Table 2), of which n = 503 also exceeded the cut-off value for the presence of SM according to FSSM. Thus, in the remaining n = 396 individuals who did not exceed the cut-off of the FSSM, there is no indication of the presence of SM, making our total sample of n = 899 a mixed sample. Inclusion criteria were that the child was between 3 and 18 years old at the time of participation and that participants had sufficient proficiency in German language. Because we collected mixed samples in each of the studies on which this study was based, no further exclusion criteria were defined. In n = 373 parents [those from study (c) and (d), as well as n = 94 parents from study (a)] we additionally collected a parent report for social anxiety (Diagnostic System for Mental Disorders According to ICD-10 and DSM-5 for Children and Adolescents, parent report for social anxiety disorder). This n = 373 individuals [age: M = 9.47 (3.23), female: 225 (60%), FSSM diagnostic score: M = 5.73 (3.52), exceeding FSSM cut-off: 197 (53%)] did not differ from the overall sample (n = 899) on sample characteristics.

**Table 1:** Sample characteristics

	Total sample
N	899
Age (3–18 years)	9.30 (4.22)
Gender (f/m)	598/301
FSSM—DS (range: 0–10)	5.62 (3.63)
Individuals who exceeded cut-off for SM on FSSM—DS (cut-off: 6 or 7 depending on age)	503 (56%)
Score of DISYPS–III FBB SOZ (the score refers to the subsample of *n* = 373 individuals who completed this questionnaire)	1.21 (.85)

*FSSM* Frankfurter Scale of Selective Mutism, *DS* diagnostic scale, *DISYPS-III FBB SOZ* Diagnostic System for Mental Disorders According to ICD-10 and DSM-5 for Children and Adolescents-III, parent report for social anxiety disorder

**Table 2:** FSSM-DS Items, paraphrased items of the DISYPS-III FBB SOZ on social fears, their node abbreviations and symptom names we use in the article

No	Description	Assigned symptom	Mean value* *M* (*SD*)
M1	Does your child fail to speak in certain situations and/or with certain individuals even though it is expected of him/her?	Silence	.70 (.46)
M2	Does he/she speak in certain situations and/or with certain individuals only very softly and tonelessly, or only use single words?	Reduced speech	.64 (.48)
M3	Is your child incapable in certain situations of shaking his/her head, of nodding or of pointing to something when asked to?	Incapacity of nonverbal communication	.45 (.49)
M4	Do his/her movements seem slow or frozen-like to you in certain situations?	Motor Inhibition	.53 (.50)
M7	Does your child lower his/her head and/or avoid eye contact in certain situations, if he/she is spoken to?	Avoidance of eye contact	.71 (.45)
M8	Does your child suppress noises (i.e., coughing, sneezing, clearing the throat, laughing, crying) in certain situations?	Suppression of noises	.41 (.49)
M9	Does your child try to avoid situations in which he/she is expected to speak by withdrawal, defiance or refusal?	Avoidance of verbal situations	.59 (.49)
M10	Is there an obvious difference between your child’s speaking behavior at home (more talkative) and outside the home (taciturn or mute)?	Selectivity of speaking behavior	.68 (.47)
S1	Marked and persistent fear of failing in performance situations (e.g., at school, in class tests, when he/she is called on in class)	Performance fear	.35 (.48)
S2	Marked and persistent fear with unfamiliar peers and trying to avoid these situations	Fear of interaction with unfamiliar peers	.44 (.50)
S3	Persistent and age unusual fear with unfamiliar or little known adults and and trying to avoid these situations	Fear of interaction with unfamiliar adults	.47 (.50)

*FSSM* Frankfurter Scale of Selective Mutism, *DS* diagnostic scale, *M1-M10* Symptoms of the questionnaire on SM (FSSM), *S1-3* Symptoms of the questionnaire on social anxiety (DISYPS-III FBB-SOZ)

*The mean values of the items M1-M10 refer to the total sample of *N* = 899, the mean values of the items S1-S3 to the subsample of n = 373 individuals for which these data were available

### Measures

#### Frankfurt scale of selective mutism

The FSSM [29] is a questionnaire assessing symptoms of SM (e.g. “Does your child fail to speak in certain situations and/or with certain individuals even though it is expected of him/her?”) in children and adolescents aged 3 to 18 years based on parent report. It is available in three development-adapted versions, namely kindergarten children aged 3 to 7 years, elementary school students aged 6 to 11 years, and adolescents aged 12 to18 years. The FSSM is freely available for research purposes. All versions include a diagnostic scale (DS) consisting of ten dichotomous items (yes–no), based on which an indication of SM can be screened for (cut-off values 6 or 7 depending on version). The ROC-analysis conducted by the authors indicates a very good differentiation between children with SM, social anxiety disorder, and children with typical development. Authors report an excellent reliability for the FSSM (Cronbach’s α = 0.90–0.98.), and comparably good reliability scores were found for the current sample (α = 0.914). The items of the DS on which the analyses of the present study are based are presented in Table 2.

#### Parent report for social anxiety disorder of the Diagnostic System for Mental Disorders (DISYPS-III FBB-SOZ)

Social anxiety was assessed using the parent rating scale for social anxiety disorder (FBB-SOZ) of the DISYPS-III [44]. This questionnaire is under license, which was obtained for the use of the questionnaire in this study. The FBB-SOZ consists of seven items on symptoms of social anxiety disorder [e.g. “Your child has a marked and persistent fear of failing in performance situations (e.g., at school, in class tests, when he/she is called on in class).”] rated on a four-point Likert scale (0 = not at all true, 3 = completely true). The authors report satisfactory to good internal consistency of Cronbach’s alpha for the scale. There is good internal consistency (α = 0.882) in the present sample.

### Study design

The present study is a secondary analysis of data on symptoms of SM and SAD based on a mixed sample of children and adolescents that we collected in four different projects. The detailed study designs of the individual projects are described in the corresponding publications: [14, 24, 27, 28, 43].

### Data analysis

#### Item selection

Since too much overlap in different symptoms leads to distortions of centrality measures [45], we selected items of the FSSM based on theoretical considerations. For this study, we selected 8 of the 10 items of the DS because they represent possible circumscribed symptoms of SM. We excluded item 5 because of its topological overlap with item 4 since both items represent a variation of motor inhibition. While item 4 describes motor inhibition in general, item 5 exclusively refers to a facial motor inhibition and is thus already covered by item 4. Furthermore, we excluded item 6 because it describes the dependence of silence on external pressure and thus describes an external symptom-inducing factor [43] rather than a symptom itself. The eight remaining items we included in the analysis are displayed in Table 2. Based on these eight symptoms, we performed the goldbricker procedure [46, 47], which checks whether two variables show a strong correlation with each other and similar correlations with other variables [46]. The goldbricker method shows the best results when the items have been preselected beforehand based on theoretical considerations [48]. Based on a minimum correlation of r = 0.70 and a threshold = 0.25, the goldbricker procedure did not indicate any redundancy.

#### Pre-analysis: network comparison of children with and without silence

To assess whether networks between children with and without the core symptom of SM are qualitatively different [38, 39], we performed a pre-analysis. For this purpose, we tested if symptom networks based on (a) children who fail to speak in specific social situations and (b) children who do not remain silent are different. If there is no qualitative difference in network structure but only quantitative difference in the strength of the links between symptoms, this implies a continuum and would warrant network analysis on the total sample of N = 899 individuals. We selected groups based on the core symptom of SM (Item 1 of DS of FSSM) for two reasons: (a) Silence lies at the heart of SM and is the only symptom described for SM in the DSM-5. Thus, the presence of silence is indicative of the presence of SM. (b) Selecting a clinical group based on a questionnaire that is later also used in the analysis is problematic. The reason is that this leads to a bias in the covariance structure and a reduction in validity, which is described in literature as Berkson's bias [49]. Therefore, we did not use cut-off values of the DS of the FSSM for group selection, since the items of the DS were object of the present network analyses. For the same reasons, we did not include item M1 in network analysis because we selected the groups on this item in the pre-analysis.

We compared networks regarding the three aspects recommended in literature [50]. (i) Network structures of both networks can be compared. Here, all edges of the networks are compared in a joint analysis to see if structures of both networks are different. (ii) Single edges can be investigated, which is only performed if the structure of the two networks (first test) is different. Here, it is tested whether the strength of individual connections between symptoms differs between the networks. (iii) The global strength of the networks can be examined, which is calculated by the absolute sum of all edges in the network. This test checks whether the networks as a whole differ quantitatively in the strength of their connectivity. Given that a comparison of networks based on binary data using groups with unequal sample sizes is associated with low power [50], we drew a random sample of n = 268 individuals from the n = 631 individuals in the group with silence. Therefore, we conducted the network comparison based on two groups of n = 268 individuals, which is sufficient for analyses with less than 10 nodes [50].

Groups (silence: n = 268, age: M = 9.50, SD = 4.25, 63.6% female, FSSM-DS: M = 7.66, SD = 2.24; no silence: n = 268, age: M = 9.82, SD = 4.07, 61.9% female, FSSM-DS: M = 1.50, SD = 2.09) only differed concerning the score of the DS (FSSM-DS: *p* < 0.001). (i) Networks of both groups did not differ significantly with regard to network structure (*p* = 0.078). Therefore, (ii) single edges were not compared regarding possible differences, as the comparison regarding network structure was not significant. (iii) The global strength differed between both networks (with silence: 17.80; without silence: 10.59; *p* < 0.001), indicating a more densely connected network in the group with silence compared to the group without. Overall, the networks (Additional file 1: Supplement A) do not differ qualitatively but only quantitatively.

#### Network analysis: symptoms of SM based on complete sample

Thus, we calculated a regulated network based on the complete sample of N = 899 individuals. Thus, our sample size is well above the minimum recommended number of included participants based on the number of nodes analyzed here [51], so we can assume sufficient statistical power. All analyses were performed using the bootnet, networktools, and qgraph packages [47, 52, 53] based on R (R Core Team, 2016). Given that symptoms were recorded using dichotomous items, the network was calculated based on an Ising model based on the eLasso algorithm [54]. For this, we used the default setup using a hyperparameter (γ) value of 0.25 as well as the AND-rule [52, 54]. Networks calculated on the basis of Ising models have edges consisting of odd ratios instead of partial correlations. However, the ratios between the edges can be interpreted in the same way as for other networks based on partial correlations. Accuracy of the network was assessed by bootstrapped difference tests between edge weights and the three different centrality measures based on a non-parametric bootstrap procedure with 1000 bootstrap samples [52]. Given that centrality measures are only to be interpreted if they can be considered stable, stability of the network was assessed using a case-dropping bootstrap procedure with 1000 bootstrap samples and subsequently calculating the correlation stability coefficient (cs-coefficient) [52]. The cs-coefficient indicates the proportion of people that can be dropped from the sample while remaining a set correlation (here 0.7) with the original network with a probability of 95%. It was recommended that the cs-coefficient should reach a value of ideally 0.5 or at least 0.25 [52]. For this paper, however, only the centrality measure strength (the sum of weight of the connections for each symptom) was considered because it is controversial how the other centrality measures can be interpreted in the context of psychopathology [55]. Additionally, we looked at node predictability; indicating the amount of variance of a single node can be explained by all other nodes [35, 36]. While centrality measures indicate the relative importance of a symptom within the network, predictability represents an absolute measure in terms of variance explained. To estimate which symptoms form a community, we used the exploratory graph analysis (EGA) [33]. The EGA combines network analysis with the walktrap algorithm, which clusters nodes within a displayed network.

#### Network analysis: symptom network of sm together with symptoms of social anxiety disorder

Given that SM and SAD show a large overlap, it is unclear which of the symptoms contained in the previously calculated network are part of the disorder entity of SM and which are more appropriately explained by social anxiety. To check whether the symptoms of the SM network are distinguishable from symptoms of SAD and form separate communities, we computed another EGA based on the subsample (n = 373) for which we had data for both a questionnaire for social anxiety (DISYPS-III FBB SOZ) and SM (FSSM). Given that we would not have had sufficient power for a joint network analysis of the previously analyzed SM symptoms and all items of the DISYPS-III FBB SOZ, we selected only the three items on social fears from this questionnaire (S1: performance fear, S2: fear of interaction with unknown peers, S3: fear of interaction with unknown adults, see Table 2). To rule out the possibility of different communities resulting from different data formats, we dichotomized the items of the DISYPS-III FBB SOZ based on information from the manual (scores 0 and 1 = not clinically relevant; scores 2 and 3 = clinically relevant). Based on the 11 dichotomous items (previous eight symptoms for SM and three symptoms for social anxiety) the goldbricker procedure did not indicate any redundancy. Furthermore, we calculated the bridge centrality (bridgestrength) of the network to investigate possible bridge symptoms that connect potential symptom clusters.

## Results

### Network of selective mutism based on the total sample

#### Network structure and communities

**Fig. 1:**
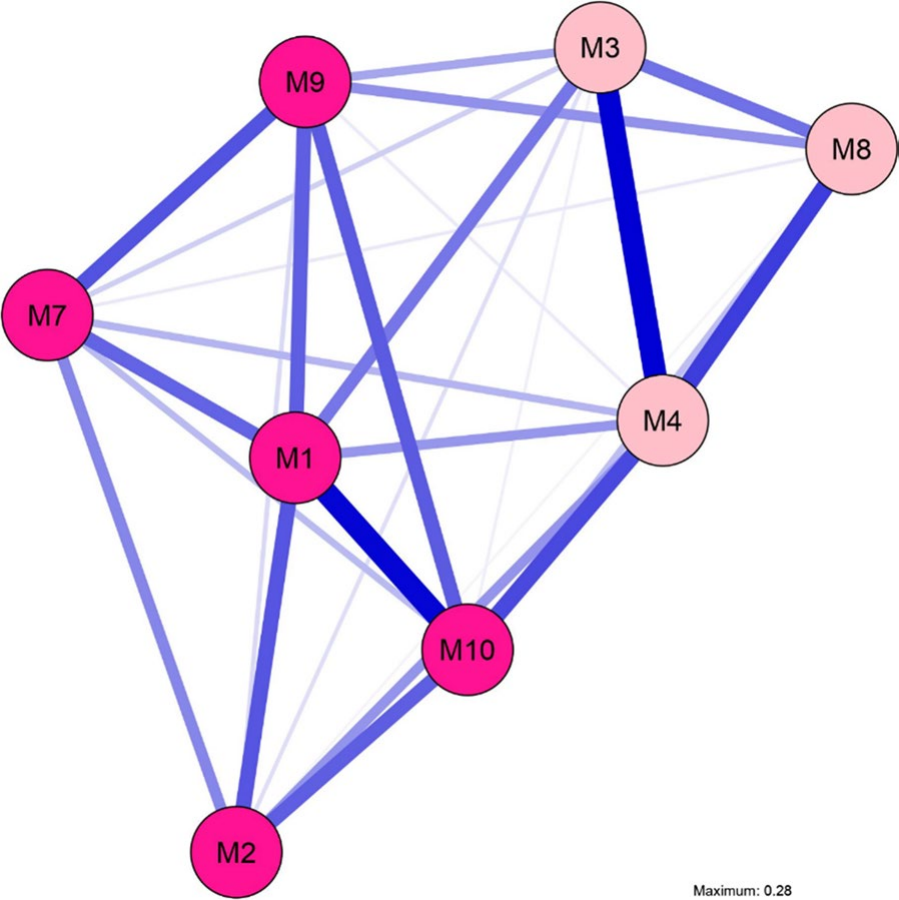
Symptom network of 8 symptoms related to selective mutism for the total sample (*N* = 899), Different colors of nodes represent different communities. Nodes represent symptoms and edges represent connections between symptoms (the thickness of the edges represents the connection strength). Included symptoms are: *M1* = silence; *M2* = reduced speech; *M3* = incapacity of nonverbal communication; *M4* = motor inhibition; *M7* = avoidance of eye-contact; *M8* = suppression of noises; *M9* = avoidance of verbal situations; *M10* = selectivity of speaking behavior

The calculated network of symptoms related to SM based on our sample of N = 899 children and adolescents is displayed in Fig. 1. All edges are positive, indicating that all symptoms are connected in an activating way and activate other related symptoms. Community analysis indicated the presence of two communities within the symptom network. The first includes the core symptom silence (M1), reduced speech (M2), avoidance of eye contact (M7), avoidance of verbal situations (M9), and selectivity of speaking behavior (M10). This community reflects both a failure of speech production and avoidance behavior. The second community, consisting of an inability to communicate nonverbally when the child is talked to (M3), motor inhibition (M4), and suppression of noises (M8), seems to reflect an affection of the motor activity.

#### Centrality of symptoms

**Fig. 2:**
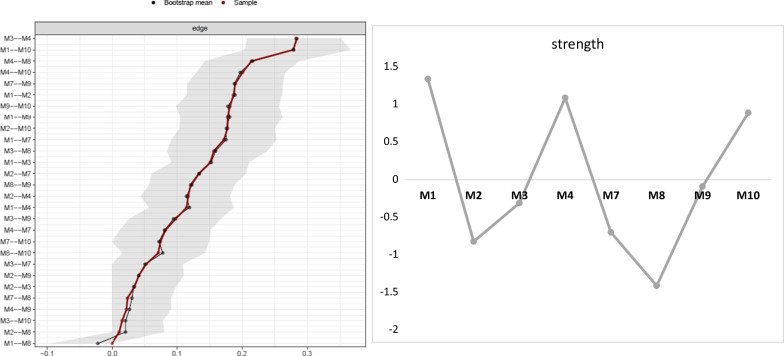
a, b Confidence intervals for every edge between nodes in networks for the total sample of *N* = 899 (Fig. 2a). The red line represents edge weights from sample mean and black line from bootstrap mean. The gray area represents the 95% confidence interval. On the left side, the respective symptom associations (i.e., M3 and M4) are plotted, for which the confidence intervals are shown. Figure 2b displays z-standardized node strength centrality for total sample. The higher the value, the higher the sum of connections a node has with all other nodes in the network. Symptoms: *M1* = silence; *M2* = reduced speech; *M3* = incapacity of nonverbal communication; *M4* = motor inhibition; *M7* = avoidance of eye-contact; *M8* = suppression of noises; *M9* = avoidance of verbal situations; *M10* = selectivity of speaking behavior between home and outside

The stability analysis shows that the network is very stable and that the strength of symptoms (cs-coefficient = 0.672) can be interpreted without restriction as it exceeded the recommended cut-off of 0.5. The strength of each symptom is shown in Fig. 2. As hypothesized, the symptom with the highest strength is the core symptom of selective mutism: silence in certain social situations (M1). The comparison of strength (see Additional file 1: supplement C) between the symptoms indicates that M1 (silence) has a significantly higher strength than all other symptoms of the network except M4 (motor inhibition) and M10 (selectivity of speaking behavior). Comparisons further reveal that M4 (motor inhibition) showed a higher strength than all other symptoms except for M1 (silence) and M10 (selectivity of speaking behavior). Additionally, M10 and M3 (incapacity of nonverbal communication) show a higher strength than M2 (reduced speech) and M7 (avoidance of eye contact). The symptoms M1 (R^2^ = 57.3%), M4 (R^2^ = 52.0%), and M10 (R^2^ = 53.40%) consistently have the highest amount of explained variance by their own edges (Additional file 1: Supplement B), also suggesting their high influence within the network. The symptoms M2 (reduced speech; R^2^ = 41.1%), M7 (avoidance of eye contact; R^2^ = 38.6%), M8 (suppression of noises; R^2^ = 33.0%), and M9 (avoidance of verbal situations; R^2^ = 43.9%) do not show higher strength compared to any other symptom, suggesting that these have a comparatively low influence within the network. The average explained variance of symptoms by all edges of other nodes is R^2^ = 45.5%.

#### Edges

Most strikingly, the edge between M1 (silence) and M10 (selectivity of speaking behavior) has a confidence interval above the range of the confidence intervals of most of the other edges (see Fig. 2a), indicating that this connection is significantly stronger than most other connections between symptoms. Furthermore, M3 (incapacity for nonverbal communication) and M4 (motor inhibition) also are strong edges that are significantly stronger than most other edges. Taken together, the connections between symptoms mentioned here seem to be particularly strong, so that these symptoms can potentially activate each other easily.

### Network of selective mutism and symptoms of social anxiety

**Fig. 3:**
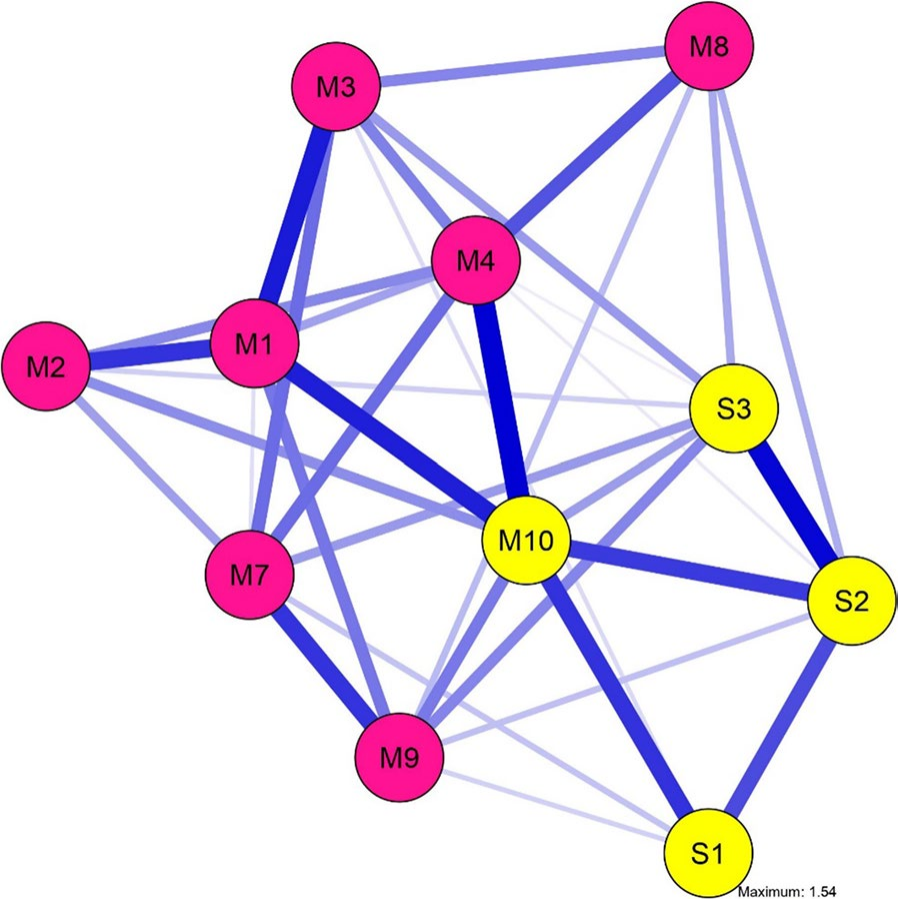
Symptom Network of 8 symptoms related to selective mutism (M1–M10) and 3 symptoms related to social anxiety disorder (S1–S3) for the subsample of *n* = 373; Nodes represent symptoms and edges represent connections between symptoms (the thickness of the edges represents the connection strength). Different colors of nodes represent different communities (M10 is the only symptom from the SM questionnaire that falls in a common community with SAD-related symptoms). All items have a dichotomous data format (symptoms of the DISYPS-III were dichotomized to ensure comparability). Included symptoms: *M1* = silence; *M2* = reduced speech; *M3* = incapacity of nonverbal communication; *M4* = motor inhibition; *M7* = avoidance of eye-contact; *M8* = suppression of noises; *M9* = avoidance of verbal situations; *M10* = selectivity of speaking behavior; *S1* = fear of performance situations, *S2* = fear of interactions with unknown children, *S3* = fear of interactions with unknown adults

The EGA based on the symptoms to SM and social anxiety resulted in the presence of two communities (Fig. 3). The first community contained all symptoms of the previously calculated SM network (which is displayed in Fig. 1) except for selectivity of speaking behavior (M10). Thus, the first community consisted exclusively of symptoms from the SM questionnaire FSSM. The second community included the symptoms performance fear (S1), fear of interaction with unknown peers (S2), fear of interaction with unknown adults (S3), and selectivity of speaking behavior (M10) and thus consists of items from both the SM- and the social anxiety questionnaire. The stability analysis shows that the network is very stable and that the bridge strength of symptoms (cs-coefficient = 0.673) can be interpreted without restriction. The symptom selectivity of speaking behavior (M10) showed the highest value of bridge strength (see Additional file 1: supplements D), which was only not significantly different from the bridge strength of the symptoms M1, M4, S2 and S3.

## Discussion

To the best of our knowledge, we have presented the first symptom network with symptoms associated with SM. We calculated the network by implementing circumscribed symptoms from a validated questionnaire on SM and based on a large mixed sample containing both children and adolescents with and without an indication for the presence of SM. In advance, we demonstrated that the symptom networks of children presented with and without silence in specific situations (the core symptom of SM) do not differ qualitatively, which allowed for network analysis in the total sample. Furthermore, we could demonstrate that the symptoms of the SM-related network can be predominantly differentiated from circumscribed social fears, thus from symptoms of SAD.

### Symptom network of SM

We aimed at investigating whether all symptoms we included in the network analysis are part of the symptom network and related to the other symptoms. The symptoms of the present network analysis are described as pathognomonic features of SM by the authors of the SM questionnaire from which the symptoms are taken [29]. Consistently, it turned out that all the symptoms we included in the calculation are part of the network, indicating that all of these symptoms potentially have relevance to the disorder of SM. According to network theory, the positively connected nodes could be mutually activating and maintaining symptoms of SM [31].

#### Symptom cluster and importance of circumscribed symptoms of SM

As expected, silence is the symptom with the highest strength and predictability, indicating that silence has a strong influence on and/or is strongly influenced by the other symptoms of the network. Given that the network is based on cross-sectional data, we cannot infer causal directions from the network at hand. Furthermore, almost all symptoms are associated with the core symptom of SM (silence). Therefore, we empirically confirmed that this symptom lies at the heart of the disorder.

We found two clusters of symptoms (communities) within the symptom network based on EGA. Symptoms form a community if they have a stronger connection to each other than to symptoms of other communities. It is important to note here that communities identified within a network by EGA can each be considered representative of a latent variable (they do not represent subgroups of individuals) [33]. In this respect, the communities in the present network seem to indicate two different variables underlying the symptoms of the mental disorder of SM. Remarkably, the symptom suppression of noises (M8) from one cluster is the only symptom not being connected to the symptom silence, which belongs to the other cluster. Therefore, the two different cluster, representing two different latent variables, may be related to two different mechanisms of silence. The first one, consisting of the incapacity to communicate nonverbally, motor inhibition, and suppression of noises, seems to fit conceptually with an anxiety-related motor inhibition, also called freezing [25]. The fear response of freezing, which is known to be mediated by the autonomic nervous system [25], is considered to affect motor activity such as movement of body parts as well as motor activity which is necessary for vocalization [56]. In this respect, this result might suggest that the freezing response is associated with reduced motor activity (including gestures) as well as the incapability to vocalize in certain situations in children with SM. In addition, the circumscribed symptom motor inhibition shows high strength and predictability within the whole SM symptom network, which also indicates the importance of frozen motor activity as a potentially important clinical feature in SM. The finding at hand of the relevance of freezing-related symptoms within the symptom network of SM is consistent with findings from recent quasi-experimental research. For example, children with SM show reduced latency to initiate gestures [23] as well as reduced visual exploration and thus probably reduced oculomotor activity [24]. Apart from the quasi-experimental studies, children with SM have been described in the literature as frozen with fear [57], and a proportion of children and adolescents with SM reported a paralyzing fear in situations that require language [14]. In this respect, the existing evidence that motor inhibition is an important clinical feature of SM is also confirmed in our network analysis due to probably forming an own cluster and showing high centrality within the whole network.

The second community, consisting of symptoms related to speech production (silence, reduced speech, selectivity of speaking behavior) and symptoms related to avoidance behavior (avoidance of verbal situations, avoidance of eye contact) seems to represent a conceptually rather heterogeneous symptom cluster. Here, interestingly, impairments in speech production are related to both symptoms associated with avoidance. In accordance with this, it has been repeatedly assumed in the past that silence in children with SM acts as an avoidance mechanism [8, 58, 59]. This connection of silence and avoidance could be supported by quasi-experimental studies based on psychophysiological measurements, which might suggest that silence in SM is associated with a reduction in physiological fear arousal [27, 60]. Overall, it is important to emphasize that the network analysis at hand is only based on subjective reports of a mixed sample, which is not sufficient in order to investigate probably biologically driven mechanisms of the inability to speak in children with SM such as freezing. Therefore, experimental research based on psychophysiological data would be needed to identify and disentangle possible mechanisms underlying the inability to speak. The symptom network at hand could, however, provide a first starting point as to which symptoms might be indicative of the possible underlying mechanisms.

Against the background that there is a subset of children with SM who do not appear to exhibit increased anxiety [10], it is important to emphasize that other symptom clusters or mechanisms not included in this symptom network may also be relevant. The symptom clusters identified here both appear to represent strongly anxiety-related latent variables (avoidance and freezing), which are consequently to some extent dependent on the symptoms included in the analysis. For this reason, it would be important in future studies to include nonanxiety-related symptoms to examine the relationship between silence and nonanxiety-related symptoms.

#### Connection between symptoms of SM

Beyond symptom clusters within the network and the importance of circumscribed symptoms, the connection between symptoms reveals information about how connected symptoms might influence each other. Remarkably, we found a strong general interconnectedness of symptoms that is reflected by the relatively high average proportion of mutually explained variance among symptoms (45.5%), which is similar to reported predictability scores of other anxiety disorders [35, 36, 42]. Given that symptom networks with a high average predictability are considered to be more strongly influenced by symptoms within the network, therapeutic interventions addressing one symptom are considered to have a strong influence on the other symptoms. Most striking is the connection between motor inhibition and incapacity to communicate nonverbally. Interestingly, the item of the FSSM regarding incapacity to communicate nonverbally does not only refer to situations with speech demands. This suggests that reduced gestures and head movements or an impaired ability to point at things (potentially also when not expected to speak) might be a clinical feature in SM closely related to motor inhibition. This may indicate that children with SM are unable to communicate nonverbally in certain social situations apart from failure to speak, which may also be associated with frozen motor activity. This inhibition might thus not be reduced to situations with expectation to speak but may occur also in social situations where no language is required. Consistently, the quasi-experimental studies also showed a prolonged movement latency [23] as well as the possible inhibition of eye movement [24] even in nonverbal social situations. Moreover, selectivity of speaking behavior shows a strong link to motor inhibition. This association also fits into the assumption that frozen motor activity might play an important role in the symptomatology of SM: Motor inhibition as a component of the fear response of freezing occurs in fear-inducing situations [25], which in SM are primarily situations outside the home [43, 61]. In this respect, it may be important for clinicians to take care of reduced motor activity that especially occurs during unfamiliar situations. Thus, considering additional symptoms in therapy of SM, such as counteracting the state of motor inhibition by motor activation, might have an additional impact on destabilizing the network and might thus contribute to the remission of SM.

### Selective mutism and social anxiety

The two symptom clusters resulting from the analysis based of both, symptoms of SM and symptoms of SAD suggest that almost all of the symptoms of the previously analyzed SM network are differentiable from symptoms of SAD. This is consistent with findings of Gensthaler et al. [29], who demonstrated that children with SM and children with SAD are well distinguishable (with large effect sizes) based on the FSSM-DSs’ sum scores. The authors refer the DSs’ high discriminatory capacity between SM and SAD to the pathognomonic features of SM contained in the scale. In contrast to this comparison of children with SM and SAD based on sum scores, we applied a network approach in the study at hand. This offers the advantage of a fine-grained analysis of which SM-related symptoms are form a different symptom cluster than symptoms of SAD and which of them fall into a common symptom cluster with SAD symptoms. Our analysis shows that only the symptom: selectivity of speaking behavior referring to an obvious difference between speaking behavior at home (more talkative) and outside the home (taciturn or mute), falls into a common cluster with the SAD-related symptoms. Given that the edges between symptoms are positive within the network, it is suggested that a large difference in speaking behavior (being mute outside home rather than at home) is strongly associated with high levels of social anxiety. Another advantage of network analysis is that it can reveal links between related mental disorders in terms of symptoms shared by different psychopathologies (bridge symptoms) [37]. Here, the symptom selectivity of speaking behavior showed the strongest connection (bridge strength) between the symptoms of both cluster and thus might be considered as a bridge symptom between SM and SAD. Consistently, the DSM-5 diagnostic criteria for SAD specify that anxiety in children may be expressed as an inability to speak in social situations [7]. Therefore, a child's failure to speak in an unfamiliar setting (e.g., therapy setting) does not necessarily equate to the presence of SM, but might also be part of (an additional) SAD. This overlap between the two disorders with respect to this symptom makes it seem even more important to consider additional symptoms in diagnosing SM, such as central symptoms of the SM network at hand (e.g. motor inhibition). While in SM it was shown that silence occurs overall less frequently at home than outside (e.g., at school) [61, 62] and that unfamiliar people and unknown places are trigger of silence [43], a proportion of affected children with SM also remains silent at home [13, 63]. Silence at home was shown to be a predictor of a poor prognosis in the few existing longitudinal studies on SM [13, 63]. Although the clusters of the symptom network does not represent subgroups, the results may be a first indication that especially silence in social situations outside the home (and consequently relatively unimpaired speech production at home) occur in children with SM and marked social anxiety. Silence at home, which seems to be associated with a poor prognosis, might not be driven by social anxiety and thus might be present in a subgroup of children characterized by different clinical features. Given that bridge symptoms are considered crucial for psychopathologies to spread [37], the presence of a great difference in speech behavior between home and out-of-home might increase the risk of developing comorbid SAD when SM is already present (or vice versa). In this context, it seems important for future research to investigate possible subgroups of SM with different symptom profiles and to examine prognosis based on prospective studies and based on directed networks that can show causal links between symptoms. Moreover, the inclusion of additional symptoms of SAD in the network (e.g., cognitions or physical symptoms) would be central to identify possible further bridge symptoms. Interestingly, other symptoms, such as avoidance of eye contact, already found in children with SAD based on quasi-experimental studies [64], were not part of the "SAD cluster." It is possible that these are similarly strongly or more strongly associated with symptoms of SM, as suggested, for example, by a previous study [24], in which avoidance of eye contact was correlated with symptom severity of SM but not with the expression of social anxiety.

Overall, the present findings may have important implications for the question of the relationship between SM and SAD. In this context, it is important to emphasize that the symptom network of SM and SAD at hand should only be seen as a first step, as important symptoms of SAD could not be included and a more comprehensive network including carefully pre-selected symptoms would be necessary for further interpretations. Nevertheless, the results of the present study might suggest that SM and SAD are two distinct entities with large overlap and common clinical features. This is indicated by symptoms of SM and SAD basically form two symptom clusters that represent two different latent variables. Given that symptoms of SM and SAD did not form one unified cluster or more cluster with mixed symptoms of SM and SAD, the results might contradict the assumption of a common disorder entity, as has been suggested by some authors [59]. In addition, none of the SAD symptoms showed any connection with the core SM symptom (silence) in the common network (Fig. 3), which also suggests that social fears (as key symptoms of SAD) are distinct from the most central symptom of SM. However, the high phenomenological overlap between SM and SAD reported in literature [8, 9] seems to be reflected in the high interconnectedness of other symptoms of SM and symptoms of SAD (see Fig. 3) and, in particular, in the potential bridge symptom of selectivity of speaking behavior.

### Strength and limitations

Our study is the first to systematically examine circumscribed symptoms of SM and the first to provide a symptom network of SM. We did this using a large sample of children and adolescents, more than half (n = 503) of whom exceeded the clinical cut-off score for SM in a screening questionnaire. In this respect, we had sufficient variance as we had numerous individuals with varying levels of SM symptomatology. Here, we were able to show that symptom networks do not differ between children with and without the given core symptom of SM, legitimizing our dimensional approach. However, there are important limitations to mention: (1) Because of Berkson's bias, we were unable to compare the networks between children meeting the cut-off value for SM on FSSM and healthy children. Although the presence of the core symptom is indicative of SM, the use of this criterion was only an approximation of a group with children meeting criteria for SM. Given that of the n = 631 children who showed the core symptom of SM, at least n = 503 children also exceeded the cut-off value for SM, this indicates a relatively good approximation. (2) It is important to note that the group comparison in the preliminary analysis, although not significant, suggests a trend difference. Although we had sufficient statistical power for the seven nodes examined, this difference could become significant based on larger sample sizes. (3) Analyses at hand are based on data from online studies that screened for SM using a validated questionnaire, so that diagnosis could not have been confirmed by a comprehensive clinical interview. To address Berkson's bias, future research should examine symptoms of SM using a clinical sample selected from a clinical interview that is not part of the network analysis. (4) Due to lack of SM questionnaires based on self-report, the symptom network of the study at hand is based solely on parent report. In this context, it is important to consider that a network of symptoms of SM that have been rated by children and adolescents themselves might differ from the present network. (5) Although we selected circumscribed symptoms of SM based on an evaluated questionnaire, we cannot be sure whether we missed important symptoms of SM that were not included in the questionnaire. This seems especially important since, by some others, SM is considered to be a heterogeneous disorder [10] and empirical links between SM and, for example, developmental delays [22] or oppositional behavior [65] have also been shown. Neglecting central symptoms may have an impact on the representativeness of network structure and centrality measures. Therefore, future research should focus on conducting a symptom network based on additional symptoms that were carefully selected in advance based on preliminary conceptual work.

## Conclusion

In the present study, we systematically examined circumscribed symptoms of SM for the first time. The results suggest that silence is appropriately viewed as the central symptom of SM, but that additional anxiety-related symptoms are also part of the disorder entity. The circumstance that both avoidance behavior and a marked fear response are included in diagnostic criteria of other anxiety disorders and are part of the present symptom network (fear response in the form of freezing) strengthens the conceptualization of SM as an anxiety disorder. Furthermore, the results suggest that two latent variables may underlie SM, possibly representing two different mechanisms of silence. Furthermore, we demonstrated that symptoms of SM could be largely distinguished from social fears, which may indicate that SM and SAD may be two separate entities. Nonetheless, the network seems to confirm that there is a large overlap (e.g., in the form of not speaking outside the home) between the two disorders.

Overall, the symptom network of the study at hand could be a good starting point for a more differentiated diagnostics and description of the disorder as well as for further research on possible mechanisms of silence. The presence of different possible mechanisms of silence or different possible symptom profiles seems to emphasize the importance of individualized interventions that address the specific mechanism. An important implication of the present study may be that it emphasizes the relevance of better understanding these mechanisms in order to develop targeted interventions. 

## Supplementary Information

**Additional file 1**: This file contains compared networks of children with silence in specific situation and children without silence in specific situations, node predicatability of total sample, strength comparison between nodes of total sample and strength comparison between nodes of subsample.

## Data Availability

The data are not publicly available due to privacy or ethical restrictions. The data that support the findings of this study are available from the corresponding author upon reasonable request.

## Abbreviations

SM: Selective mutism
SAD: Social anxiety disorder
FSSM: Frankfurt Scale of Selective Mutism
DS: Diagnostic scale of FSSM
SMQ: Selective Mutism Questionnaire
DISYPS-III: Diagnostic system for mental disorders according to ICD-10 and DSM-5 for children and adolescents
FBB-SOZ: Parent report for social anxiety disorder of DISYPS-III
EGA: Exploratory graph analysis
