# Supplementary material for: Silence and related symptoms in children and adolescents: a network approach to selective mutism
Source: BMC Psychol. 2022 Nov 16;10:271. doi: 10.1186/s40359-022-00956-9 (PMC9670669; doi:10.1186/s40359-022-00956-9)
Supplement: Supplementary file 1 — Additional file 1: This file contains compared networks of children with silence in specific situation and children without silence in specific situations, node predicatability of total sample, strength comparison between nodes of total sample and strength comparison between nodes of subsample. [file 40359_2022_956_MOESM1_ESM.docx]

**Supplements**


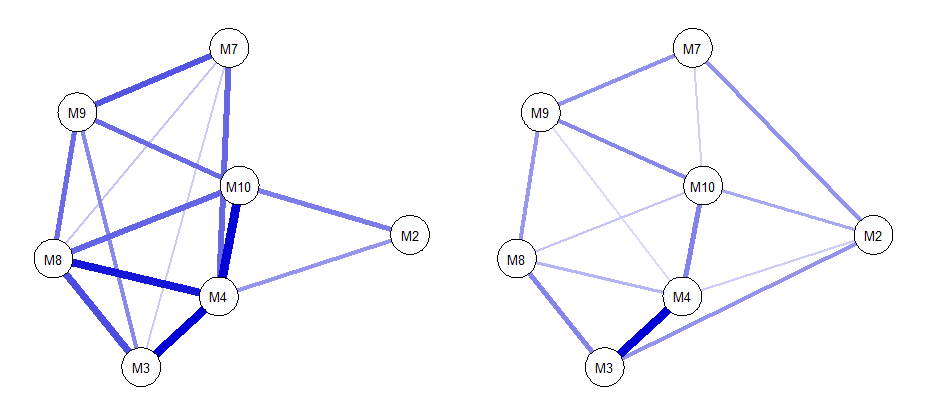


2)

1)

**Supplement A**

Compared networks of 1) children with silence in specific situation (n = 268) and 2) children without silence in specific situations (n =268). In order to compare networks of identical sample sizes, we drew a random sample of n = 268 of children with silence (n = 631). Included symptoms: *M2* = reduced speech; *M3 =* incapacity of nonverbal communication; *M4* = motor inhibition; *M7* = avoidance of eye-contact; *M8* = suppression of noises; *M9* = avoidance of verbal situations; *M10* = selectivity of speaking behavior; *Symptom M1* (silence) could not be included as we selected groups based on this item.

**Supplement B**: Node Predicatability of total sample, N = 899

| Item | R² |
| --- | --- |
| M1 | .573 |
| M2 | .411 |
| M3 | .449 |
| M4 | .520 |
| M7 | .386 |
| M8 | .330 |
| M9 | .439 |
| M10 | .534 |


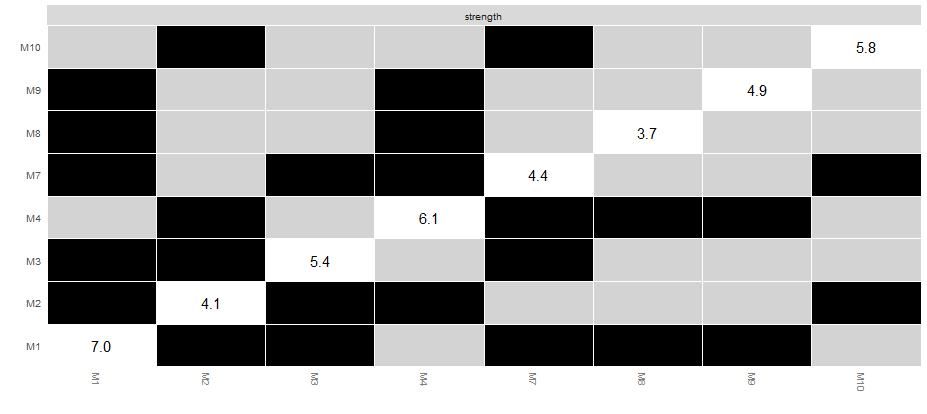
 **Supplement C**: Strength comparison between nodes of total sample N = 899; black box = significant difference; Included symptoms: *M1* = silence; *M2* = reduced speech; *M3 =* incapacity of nonverbal communication; *M4* = motor inhibition; *M7* = avoidance of eye-contact; *M8* = suppression of noises; *M9* = avoidance of verbal situations; *M10* = selectivity of speaking behavior


**Supplement D**

Strength comparison between nodes of subsample n = 373; black box = significant difference; and bridge strength values of symptoms of symptoms related to selective mutism (M1-M10) and 3 symptoms related to social anxiety disorder (S1-S3). Included symptoms: *M1* = silence; *M2* = reduced speech; *M3 =* incapacity of nonverbal communication; *M4* = motor inhibition; *M7* = avoidance of eye-contact; *M8* = suppression of noises; *M9* = avoidance of verbal situations; *M10* = selectivity of speaking behavior; *S1* = fear of performance situations, *S2* = fear of interactions with unknown children, *S3* = fear of interactions with unknown adults
